# Sampling highbush blueberries for *Rhagoletis mendax* (Diptera: Tephritidae) reveals shifting activity timing and cultivar susceptibility

**DOI:** 10.1093/jee/toaf219

**Published:** 2025-09-15

**Authors:** Steven Van Timmeren, Rufus Isaacs

**Affiliations:** Department of Entomology, Michigan State University, East Lansing, MI, USA; Department of Entomology, Michigan State University, East Lansing, MI, USA

**Keywords:** IPM, phenology, integrated pest management, *Drosophila suzukii*, blueberry maggot

## Abstract

The blueberry maggot, *Rhagoletis mendax* Curran (Diptera: Tephritidae), is a native pest of blueberries in eastern North America. Invasion by spotted-wing drosophila, *Drosophila suzukii* (Matsumura) (Diptera: Drosophilidae), has caused increased use of insecticides during *R. mendax* activity resulting in control of both pests, however recent detections of early-season infestation by *R. mendax* suggests a changing phenology. Monitoring of adult flies over multiple years revealed *R. mendax* activity shifting approximately 3 wk earlier in the season at times when *D. suzukii* activity is low. Larval sampling in infested blueberry fields over 11 yr showed a similar changing pattern of activity. Berries collected from a multicultivar planting over 3 yr revealed that *R. mendax* laid eggs most often in ripening fruit and can even lay eggs in green fruit, indicating risk of infestation before *D. suzukii* infestation risk. Significantly more *R. mendax* were detected in later ripening cultivars than earlier cultivars, reflecting overlap between ripening stage and fly activity. When collected pupae were placed in non-overwintering conditions, approximately 1% emerged as adults within the first 57 d, showing that non-diapausing individuals have potential to also extend activity late in the season. The results presented here suggest that *R. mendax* can adapt to competition from invasive competitors, although the risk of fruit infestation remains highly influenced by overlap between flight activity and berry ripening.

## Introduction

*Rhagoletis mendax* Curran (Diptera: Tephritidae), the blueberry maggot, is native to eastern North America and has been reported as a major pest of highbush blueberry (*Vaccinium corymbosum* L.) and lowbush blueberry (*Vaccinium angustifolium* Aiton) for more than a century (Woods 1914, Patch and Woods 1922). Fly larvae feed on ripe blueberries and when fruit drop off the bush the larvae burrow into the soil to pupate and spend the winter before emerging as adults the following season. Integrated pest management programs have managed this pest using yellow sticky traps baited with ammonium acetate to capture adult flies when they first emerge (Rodriguez-­Saona et al. 2015). Typically, insecticide applications to protect fruit from infestation are initiated when adult flies are trapped (Gaul et al. 2002). For most of the past century, growers could protect their blueberries using 1 or 2 insecticide applications in a season (Wood et al. 1983, Gaul et al. 1995). The invasion by *Drosophila suzukii* (Matsumura) (spotted-wing drosophila) more than a decade ago caused growers to switch to more frequent insecticide applications beginning when the fruit ripen (Van Timmeren and Isaacs 2013, Tait et al. 2021). The increased focus on *D. suzukii* control quickly overshadowed the importance of *R. mendax* as a pest in commercial blueberry fields. Modeling of *R. mendax* populations suggests it may exhibit changes to its phenology (Teixeira and Polavarapu 2001, Shope et al. 2023), highlighting the importance of remaining vigilant regarding this pest. Changes in *R. mendax* phenology in relation to fruit infestation could be caused by increased insecticide use, changes in diapause induction, the shifting complex of cultivars that has expanded the time when berries are available for oviposition, climate-induced changes in ripening, and competition with other insects that infest ripe blueberries.

Rebuilding IPM programs after arrival of *D. suzukii* needs to incorporate other key insect pests to be successful. The flexibility of pest species’ phenology is an important component of this, since pest timing can change over time. Teixeira and Polavarapu (2001, 2002) showed that *R. mendax* can shift emergence to later in the season based on host fruit ripening time. More recently, Shope et al. (2023) found captures of adult *R. mendax* in New Jersey blueberry farms have decreased and shifted to later in the season after *D. suzukii* invaded the region, a trend the authors hypothesized was due to *D. suzukii* larvae outcompeting *R. mendax* in blueberries. Research on a closely related species, *R. pomonella* (Walsh), has shown adaptation of populations to predictable host plant ripening phenology where genomic variability has resulted in shifts in diapause length and emergence (Powell et al. 2014, Doellman et al. 2018, Meyers et al. 2020, Calvert et al. 2022).

Highbush blueberry farms in the eastern United States typically include several cultivars to allow for harvest from early summer to late summer (June to September). These cultivars can vary in their inherent susceptibility to insect pests and in their risk of pest infestation due to the relative phenology of the ripening and the pest activity. Comparison of 18 *V. corymbosum* cultivars in Rhode Island found significantly fewer *R. mendax* larvae in early-ripening cultivars such as ‘Earliblue’ and ‘Bluetta’ than in later cultivars including ‘Bluehaven’ and ‘Coville’ (Liburd et al. 1998), a pattern that lined up with the adult fly oviposition activity (late-July to early August). So far this is the only study investigating blueberry cultivar susceptibility to *R. mendax*. Since this occurred prior to *D. suzukii* invasion of North America, it is unknown how *R. mendax* infestation patterns may have changed now that there are 2 blueberry-infesting flies in eastern North America.

The connection between crop phenology and insect activity is affected by the duration and intensity of diapause, which can influence insect activity periods. Historically, *R. mendax* has been reported as having one generation per year (Lathrop and Nickels 1932, Rodriguez-Saona et al. 2015) except in northern regions where adult emergence can take 2 to 4 yr (Lathrop and Nickels 1932, Boller and Prokopy 1976, Neunzig and Sorensen 1976). However, Payne and Berlocher (1995) cite unpublished data where 1 adult fly emerged out of 68 pupae that had been placed in laboratory conditions. Likewise, Dambroski and Feder (2007) cite unpublished data where 5% of *R. mendax* pupae reared from blueberries collected in Michigan eclosed as adults without undergoing diapause. Beyond these brief mentions, there is little information on non-diapausing adult emergence in *R. mendax*, despite evidence in other *Rhagoletis* species (Calvert et al. 2022). This includes *R. pomonella*, which has been shown to exhibit a second generation of adults (Pettit 1926, Porter 1928, Boller and Prokopy 1976) with the second generation able to develop to pupation in southern portions of its range (Dolphin et al. 1970, Powell et al. 2014). Other *Rhagoletis* species, including *R. completa* (Cresson) (Rull et al. 2019), *R. cornivora* Bush (Smith 1985), *R. indifferens* Curran (Frick et al. 1954), and *R. zephyria* Snow (Schwarz et al. 2007, Yee et al. 2021) also have a small proportion of adults emerging as a second generation late in the season. Understanding whether *R. mendax* has continued development late in the season in some circumstances will be important given the recent extension of the blueberry growing season facilitated by breeding programs (Edger et al. 2022) and increasing temperatures that provide longer growing seasons in the Great Lakes region (Pryor et al. 2014).

Shifting timing of *R. mendax* emergence could also be associated with altered oviposition behavior of adult flies after emergence. Adult *R. mendax* are assumed to lay eggs into almost-ripe or ripe fruit (Rodriguez-Saona et al. 2015), but little research has focused on this (Stelinski et al. 2004). Studies on other *Rhagoletis* species indicate that green fruit are less preferred for oviposition and ripening and ripe fruit are generally preferred (Boller and Prokopy 1976, Messina et al. 1991). However, some *Rhagoletis* species are capable of laying eggs in unripe fruit, including *R. indifferens* in green sweet cherries (Frick et al. 1954) and *R. lycopersella* Smyth in *Lycopersicum pimpinellifolium* (Smyth 1960). Understanding the timing of *R. mendax* oviposition may provide insights into the risk period for blueberries that can be incorporated into management programs.

The goal of this study was to investigate *R. mendax* phenology, patterns of infestation in different blueberry cultivars, and the degree of diapause in fields where *D. suzukii* has become the dominant economic pest. To do this we included the following objectives: (i) determine patterns of infestation and activity by *R. mendax* and *D. suzukii* in Michigan blueberry fields, (ii) test whether *R. mendax* exhibits non-diapausing adult emergence, (iii) compare *R. mendax* infestation in different cultivars of highbush blueberry, and (iv) quantify the relationship between fruit ripeness and oviposition behavior of *R. mendax*.

## Materials and Methods

### Patterns of Berry Infestation and Activity

Unsprayed and minimally sprayed ‘Jersey’ blueberry fields in west Michigan were monitored every growing season from 2013 to 2023. Four to 6 sites were monitored each year except for 2 yr where additional sites were monitored (9 sites in 2017 and 8 in 2018). To monitor for *R. mendax* and *D. suzukii* infestation, ripe blueberry samples (∼114 ml per sample, ∼60 berries) were collected once a week from the border of each field where infestation is usually higher (Rodriguez-Saona et al. 2015, Levenson et al. 2025). Fruit samples were collected beginning when ripe fruit were first present (early/mid-July) and continuing until ripe fruit were no longer available (mid/late September). One to 4 samples were collected at each visit and *R. mendax* and *D. suzukii* larvae were detected using the filter larval monitoring method (Van Timmeren et al. 2017, 2021, Van Timmeren and Isaacs 2024). Briefly, blueberries were weighed and then lightly squeezed, placed in a 8.25% (w/v) salt water solution for 1 h, after which the liquid was sifted through a reusable coffee filter. The total number of *R. mendax* and *D. suzukii* larvae collected in the coffee filter was counted under a stereomicroscope (Olympus SZX10 set at 5x magnification [10x eyepiece lens, 0.5x objective lens], Olympus America, Inc., Center Valley, Pennsylvania, United States). At sites where more than one sample was collected on a particular sampling date, we combined the sample data for each week’s sampling. Weekly *R. mendax* and *D. suzukii* infestation data were each averaged across sites for the earlier sampling years (2013 to 2019) and the recent sampling years (2020 to 2023).

In addition to the larval sampling from 2013 to 2023, more detailed sampling was conducted at 5 unsprayed blueberry sites in 2024 to determine the numbers of *R. mendax* and *D. suzukii* reared from blueberries over the season. Fruit samples were collected from 2 ‘Jersey’ fields at each site on 5 dates spread across the time when ripe fruit were available (9 July, 18 July, 24 July, 31 July, and 8 August). Four separate 114 ml fruit samples were collected from each field border for a total of 8 fruit samples from each site on each date. Samples were placed in containers using the rearing methods described in Van Timmeren et al. (2025), with berries placed in stainless steel wire mesh holders placed over 4 oval cotton pads inside square plastic containers (12 × 12 × 8 cm) along with 2 yellow sticky traps (3.8 × 12.7 cm). Rearing containers were kept at 23 to 24 °C and 70% to 80% relative humidity on a 16:8 d:night cycle for at least 21 d at which point the containers were assessed and the total number of *D. suzukii* adults and *R. mendax* pupae, dead larvae, and adult flies were counted. Cotton pads in the containers were taken apart during the assessment process as larvae would sometimes pupate inside the pads.

To determine phenology of adult *R. mendax* flies, yellow sticky traps (Trécé Pherocon AM No-Bait, Great Lakes IPM, Vestaburg, Michigan, United States) baited with a Polycon ammonium acetate lure (Great Lakes IPM) were deployed in a blueberry planting at the Trevor Nichols Research Center (TNRC) in Fennville, Michigan. This planting consists of ∼6 acres of mature ‘Bluecrop,’ ‘Blueray,’ and ‘Jersey’ bushes as well as a replicated cultivar planting with 6 cultivars (‘Aurora,’ ‘Bluecrop,’ ‘Duke,’ ‘Elliott,’ ‘Jersey,’ and ‘Liberty’) that was used for the cultivar comparison in this study. These bushes were planted in 2010 to replace mature ‘Bluecrop,’ ‘Jersey,’ and ‘Rubel’ and a mixed cultivar planting. Three traps were set up in one of the blocks every year from 2008 to 2024 (2008 to 2009: ‘Rubel,’ 2010 to 2024: ‘Bluecrop’) and traps were checked weekly from mid-late June to late August each year. The number of adult *R. mendax* per trap was averaged for each sampling date. In addition to trapping *R. mendax* adults, *D. suzukii* adults were trapped in a ‘Jersey’ block at TNRC from 2020 to 2024 using yeast-sugar traps as described in Van Timmeren and Isaacs (2013). The 2008 to 2013 period included the years before *D. suzukii* was present or early in their invasion and was during the re-establishment of the bushes at this site. We excluded 2010 to 2011 as no blueberries were available due to the bushes growing during establishment. The *R. mendax* captures from these early years were compared to the more recent trap data when *D. suzukii* was well established (2014 to 2019 and 2020 to 2024). The early years and most recent *R. mendax* trap data were compared to the *D. suzukii* trap data by comparing peak adult capture dates in each year. Data from the 2014 to 2019 time period were omitted from this analysis due to very low adult *R. mendax* captures in those years.

In 2023, observations of adult flies on blueberry bushes were conducted to determine adult activity during the primary fruiting period. Observations were conducted in a mixed cultivar planting at TNRC containing 6 common blueberry cultivars (‘Aurora,’ ‘Bluecrop,’ ‘Duke,’ ‘Elliott,’ ‘Jersey,’ and ‘Liberty’) planted in 36 12-bush rows (6 total replicates). Two observers slowly walked down either side of each blueberry row and recorded all *R. mendax* adults visible on the bushes, taking ∼1 min to walk the length of each row. Assessments took place on 8 different dates (11 July, 14 July, 17 July, 18 July, 19 July, 1 August, 2 August, and 3 August) between 09:30 and 15:30 h when the adult flies were most active (Smith and Prokopy 1981), and observations were not conducted on cool days or days with rain. The total number of adult flies was recorded on all bushes in each row and the values from each of the 6 rows within each replicate was combined to obtain the total number of flies observed per replicate. These values were used to calculate the average number of *R. mendax* observed on each sampling date.

### Emergence Study

To quantify the emergence of *R. mendax* adults that eclosed without diapause, larvae were reared out of blueberries collected in July and August 2021, 2022, and 2023 from unsprayed, minimally sprayed, or organically managed blueberry fields in southwest Michigan. Fruit were placed in plastic containers as described previously to allow larvae to complete development, exit the fruit, and pupate on or in the cotton pads at the bottom of the containers. Pupae were subsequently collected from rearing containers, and placed in plastic soufflé cups (57 or 92 ml Solo P200N and Solo P325N, Dart Container Corporation, Mason, Michigan) which were then incubated in an environmental chamber at 22 to 24 °C and 78% to 88% relative humidity on a 16:8 d:night cycle. The cups were checked for emerging adults every ∼1 to 2 d for the first ∼50 to 60 d after being put in the cups, then checked 1 to 2 times per week for the next ∼30 d, followed by sporadic checks until 174 d after fruit were placed into rearing containers (∼January to March of the following year).

Adult emergence data were grouped based on categories established from previous studies on *Rhagoletis pomonella* (Dambroski and Feder 2007, Toxopeus et al. 2021, McIntyre et al. 2023). In these studies, adults that eclosed within 36 d of pupation were considered non-diapausing, those that eclosed 37 to 65 d after pupation were considered to have entered a weak diapause, and those that eclosed more than 65 d after pupation were considered to have entered a full diapause. Because pupae in this study were not collected from containers immediately after pupal formation, pupal age was estimated by counting 21 d from when the berries were placed in rearing containers. The 21 d estimate was based on the 17 to 30 d required for full development from egg to the point of pupation (Rodriguez-Saona et al. 2015). Adding this estimate, emerging adults in this study were grouped into 1 of 3 categories: 1 to 57 d, 58 to 86 d, or 87 to 174 d after blueberries were placed in rearing containers.

### Infestation in Different Cultivars

To better understand patterns of infestation in different blueberry cultivars, a study was conducted by collecting ripe fruit samples from each of the 6 cultivars in the planting described above (‘Aurora,’ ‘Bluecrop,’ ‘Duke,’ ‘Elliott,’ ‘Jersey,’ and ‘Liberty’). A filter test was used to assess the fruit for presence of *R. mendax* larvae. Ripe blueberries (50 to 114 ml per sample) were collected from each cultivar plot on 2 sampling dates in 2021 (2 August, 10 August), one date in 2022 (26 July 2022), and one date in 2023 (3 August 2023). We also assessed infestation by collecting fruit samples and placing them in rearing containers to allow *R. mendax* larvae to pupate. Samples (∼25 to 140 ml per sample depending on fruit availability) were collected from the TNRC cultivar planting in 2023 (10 July, 28 July, 15 August) and 2024 (18 July). Blueberries were collected from each of 4 ripeness categories: late green (no coloration yet), trace color (blue color just starting to show on the berry), ripening (∼25% to 74% of the berry turned blue), or ripe (75% to 100% of the berry turned blue). In 2022 the blueberries were placed on 4 oval cotton pads in 455 ml clear plastic containers along with a yellow sticky trap (3.8 × 12.7 cm, Olson Products, Inc., Medina, Ohio, United States) to capture emerging *D. suzukii* and *R. mendax* adults. Container lids were ventilated by gluing 150 micron mesh (The Cary Company, Addison, Illinois, United States) over a 5 cm diameter hole made in the lid. Rearing samples in 2023 and 2024 used the square plastic containers as described previously (Van Timmeren et al. 2025). In all years, after at least 21 d the containers were assessed for *R. mendax* pupae, dead larvae, and adult flies.

Seasonal berry ripening phenology for the 6 cultivars in the replicated planting was determined by assessing 5 clusters in each of 3 replicates. Berries were assessed 2 to 3 times per week from early June through the end of August each year from 2013 to 2023. All blueberries on each cluster were classified into one 6 ripeness categories: early green, late green, trace coloring, 25% to 50% of the berry was blue, 51% to 75% blue, and 76% to 100% blue.

### Fruit Ripeness Preference

Fruit rearing data from the previous experiment were used to determine if *R. mendax* oviposition is affected by blueberry ripeness and if oviposition choices change over time. We also compared oviposition by wild *R. mendax* across cultivars and ripeness stages in 2023 by exposing blueberries at the TNRC cultivar planting described above for 1-wk intervals to allow flies to oviposit on the recently exposed berries. To accomplish this, individual blueberry canes were covered with mesh paint strainer bags (The Cary Company, Addison, Illinois, United States) immediately after bloom to prevent insects from accessing the blueberries on the covered canes. Each covered cane contained multiple blueberry clusters from a single cultivar. Enough canes were covered to provide 2 sets of 4 replicates for each of the 6 cultivars (‘Aurora,’ ‘Bluecrop,’ ‘Duke,’ ‘Elliott,’ ‘Jersey,’ and ‘Liberty’). One set of mesh bags was removed from canes on 24 July 2023 and berries were left exposed for 7 d at which point all fruit on the exposed canes were harvested. This was repeated with the second set of bagged canes where bags were removed on 31 July 2023 and berries were harvested 7 d later. Harvested berries on each date were sorted into the same 4 ripeness categories mentioned previously (late green, trace color, ripening, ripe) and placed in rearing containers as described above. These were incubated for at least 21 d at which point the total number of *R. mendax* pupae, dead larvae, and adult flies were counted. Any pupae that were present were placed in souffle cups and used for the emergence study described previously.

### Statistical Analyses

Statistical analyses were conducted using Systat 13 (Systat Software, Inc., Chicago, Illinois, United States). Data were tested for normality using a Shapiro-Wilk test and for homogeneity of variance using Levene’s test. All mean values are presented ± SE, and an alpha value of 0.05 was used for all analyses. To compare detection of larvae over the course of the season from the multi-year larval sample in blueberry fields, the total number of *R. mendax* or *D. suzukii* larvae were grouped by month within each year. The data on total number of larvae per 100 g of blueberries for the 3 primary sampling months (July, August, and September) were compared across all years using a Kruskal-Wallis test followed by a Conover-Inman test for post hoc analysis. An additional analysis was conducted to compare the total number of larvae detected in the earlier (2013 to 2019) and recent (2020 to 2023) sampling years. Weekly averages were used to obtain a season average for each year. Two-sample *t*-tests were then used to compare early and recent years for both *R. mendax* and *D. suzukii*.

To compare *R. mendax* reared from blueberries collected at different time periods in the more intensive field sampling in 2024, the number of flies reared per 100 g of fruit were log (X + 1) transformed and analyzed using analysis of variance (ANOVA) with time period as the fixed effect. *Drosophila suzukii* rearing data were analyzed using a similar approach except these data did not require transformation before analysis. A Tukey’s honestly significant difference (HSD) test was used for means separation. For the adult *R. mendax* and *D. suzukii* trapping at TNRC, peak capture dates for each year were converted to a day of the year value and the values from *R. mendax* historical trapping years (2008 to 2009, 2012 to 2013) were compared to recent trapping years for *R. mendax* and *D. suzukii* (2020 to 2024) using ANOVA where *R. mendax* trapping period (historical, recent) and *D. suzukii* trapping (recent) were fixed effects. A Tukey’s HSD test was used for means separation. The total numbers of adult *R. mendax* observed per replicate were compared across all dates using a Kruskal–Wallis test followed by a Conover-Inman test for post hoc comparisons.

To compare cultivar susceptibility, the total number of *R. mendax* larvae per 100 g of blueberries were analyzed using ANOVA with cultivars as fixed effects and followed by Tukey’s HSD test for means separation. Data in 2021 (2 August sample only) and 2023 were log (X + 1) transformed to achieve normality prior to analysis. Data from ‘Duke’ bushes were only included in the analysis in the 2023 sample as there were fewer than 3 replicates in each of the previous 2 yr.

For fruit samples collected to rear out *R. mendax* and compare infestation among cultivars and fruit ripeness stages, the total numbers of *R. mendax* per 100 g of blueberries were not normally distributed even after data transformation. Consequently, they were analyzed using a Kruskal-Wallis test followed by a Conover-Inman test for post-hoc analysis (3 or more treatments) or a Mann-Whitney *U* test (2 treatments). Data were analyzed to compare ripeness stage within each cultivar and were also analyzed to compare cultivar within each ripeness stage. In a few cases, a lack of available fruit resulted in fewer than 3 replicates so these were excluded from the analyses. Data from the 2 sets of bagged canes in 2023 were analyzed to compare ripeness stage and cultivar in the same manner as for the rearing data. To compare the number of *R. mendax* reared out between the 2 bagged canes dates, the total number of *R. mendax* larvae per 100 g of fruit were compared for all cultivars within each ripeness stage category using a Mann-Whitney *U* test. Only cultivars where data were available from both sets of bagged canes were included in the analysis.

## Results

### Patterns of Infestation and Activity in Blueberry Fields

**Fig. 1.**
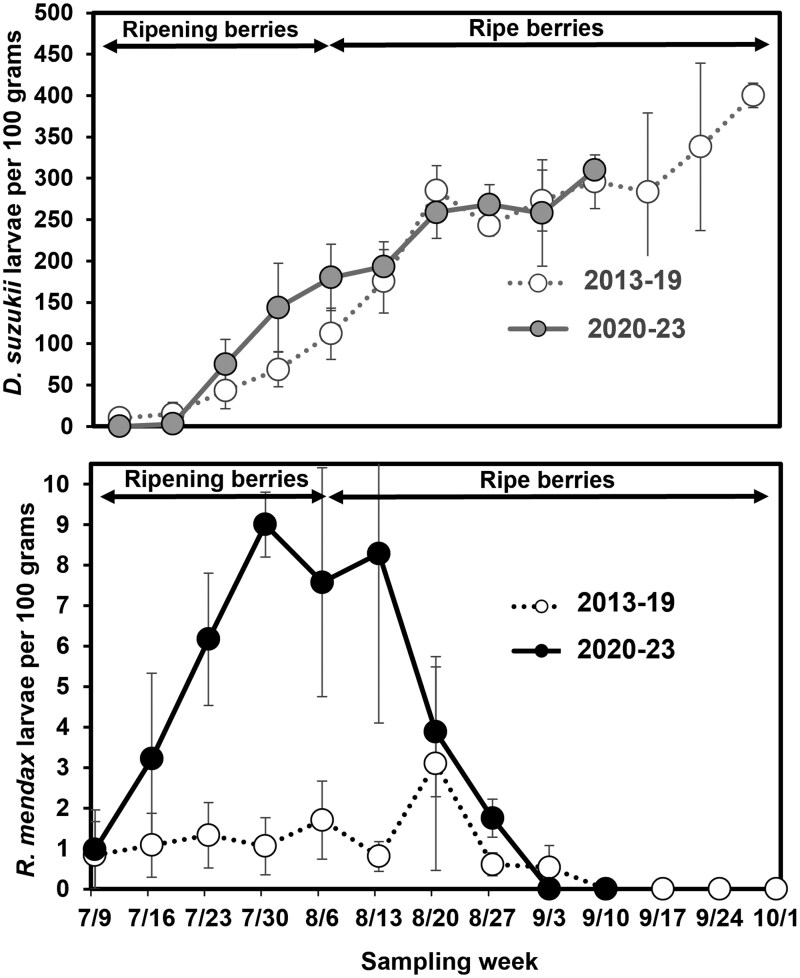
The average total number of *Drosophila suzukii* larvae (top graph) and *Rhagoletis mendax* larvae (bottom graph) per 100 g of blueberry. Ripe fruit samples (∼114 ml per sample) were collected weekly from unsprayed and minimally sprayed ‘Jersey’ fields in each of 11 yr (2013 to 2023) and assessed for larvae using a filter test. Weekly samples for all sites were averaged for the 2013 to 2019 time period and the 2020 to 2023 time period and averages are presented ±SE. Ripening phenology of berries at the sites are listed at the top of the graph; the ripe berry timing listed is when at least 50% of assessed berries were ripe.

Over the 11 yr of monitoring *R. mendax* and *D. suzukii* larvae in unsprayed and minimally sprayed blueberry fields, larvae were usually detected soon after the first ripe blueberries were collected in early July (Fig. 1). Detection of *R. mendax* larvae increased rapidly in July and remained steady in August before declining into September. In comparison, detection of *D. suzukii* larvae started low and increased steadily through the entire season. When comparing larvae detected in each month, significantly fewer *R. mendax* larvae were detected in September than in either July or August (*H* = 8.51, df = 2, 26, *P* = 0.014) and significantly fewer *D. suzukii* larvae were detected in July than in either August or September (*H* = 16.98, df = 2, 26, *P* < 0.001). Significantly more *R. mendax* larvae were detected in the recent years than in the earlier years (2013 to 2019: 1.37 ± 0.73 *R. mendax* per 100 g, 2020 to 2023: 5.7 ± 1.02; t = −3.15, df = 1, 9, *P* = 0.012), whereas there were no significant differences between earlier and recent years for *D. suzukii* (2013 to 2019: 121.12 ± 17.39 *D. suzukii* per 100 g, 2020 to 2023: 159.95 ± 16.89; t = −1.60, df = 1, 9, *P* = 0.18).

For the more detailed sampling at 5 unmanaged blueberry sites in 2024, more *R. mendax* were reared from blueberries on 9 July (5.75 ± 2.13 *R. mendax* per 100 g) and 18 July (5.72 ± 1.74) than the last 3 time periods (24 July: 4.04 ± 0.43, 31 July: 2.95 ± 0.94, 8 August: 1.4 ± 0.86), though these differences were not significant (*F* = 2.84, df = 4, 20, *P* = 0.051). At the 8 August sampling period, 3 of the 5 sites had no *R. mendax* emerge from any of the collected samples. *Drosophila suzukii* larvae were less abundant in the first time period than any subsequent collections (9 July: 106.56 ± 30.34 *D. suzukii* per 100 g, 18 July: 289.94 ± 26.73, 24 July: 282.05 ± 32.96, 31 July: 281.88 ± 27.72, 8 August: 306.57 ± 16.30; *F* = 9.11, df = 4, 20, *P* < 0.001).

**Fig. 2.**
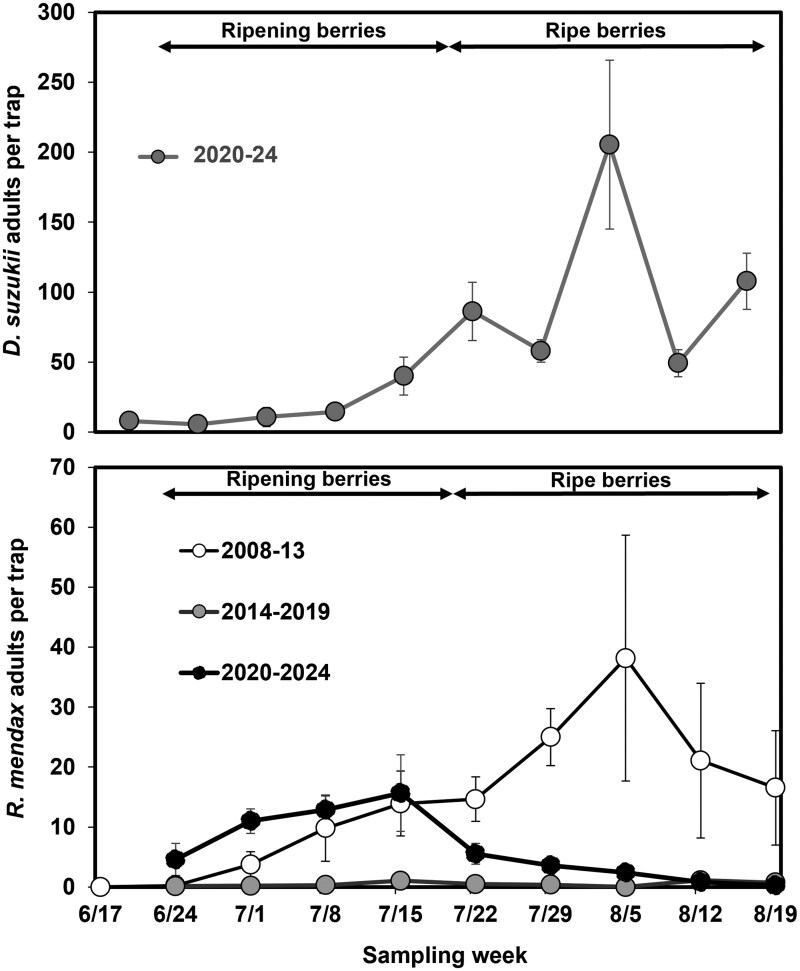
The average total number of *Drosophila suzukii* adults per trap (top graph) and *Rhagoletis mendax* adults per trap (bottom graph) in traps placed in a blueberry field at the Trevor Nichols Research Center in Fennville, Michigan. Three *R. mendax* traps were deployed from mid-June to late-August each year from 2008 to 2024 and one *D. suzukii* trap was deployed from mid-June to late-August during the 2020 to 2024 time period. All traps were checked weekly and capture of adult flies was averaged for 3 time periods: early years (2008 to 2013), middle years (2014 to 2019), and recent years (2020 to 2024). Two years were excluded (2010 to 2011) due to blueberry fields being replanted in these years. Ripening phenology of berries at the sites are listed at the top of the graph; the ripe berry timing listed is when at least 50% of assessed berries were ripe.

Adult *R. mendax* were typically detected first at the TNRC blueberry planting in early July. During the historical sampling (2008 to 2009, 2012 to 2013), capture of adults increased steadily until peaking in early August, while in recent years (2020 to 2024) the captures peaked in mid-July before declining (Fig. 2). Capture of *D. suzukii* adults followed a similar pattern to the historical *R. mendax* trapping, with few adults caught in early July with a steady increase until a peak in early August. The average day of the year when the peak of adult *R. mendax* was detected was almost 3 wk earlier in recent years than in historical years and more than 3 wk earlier than the *D. suzukii* peak (historical *R. mendax*: 214 ± 6.8 d of the year, recent *R. mendax*: 193.6 ± 3.5, recent *D. suzukii*: 225 ± 4.4; *F* = 11.7, df = 2, 11, *P* = 0.002).

Adult *R. mendax* flies were detected on blueberry bushes on all July sampling dates in 2023 (11 July: 3.0 ± 1.0 adults per replicate, 14 July: 7.2 ± 3.4, 17 July: 4.2 ± 1.4, 18 July: 8.3 ± 4.3, 19 July: 5.0 ± 2.1), wheras none were detected on any of the 3 August sampling dates (1, 2, 3 August). The 2 mo were highly significantly different (*H* = 19.85, df = 7, 25, *P* = 0.006).

### Emergence Study

Just under 6,000 *R. mendax* pupae were collected and placed in soufflé cups then observed for emergence over the 3 yr of the study (2021: 1666, 2022: 2303, 2023: 1943). In 2021 and 2022, 1.2% and 1.3% of pupae had adults emerge within the first 57 d after fruit collection, but in 2023 this decreased to 0.1%. In the 58 to 86 d time period after fruit collection, adult emergence was 1.2% (2021), 2.4% (2022), and 0.3% (2023). During the 87 to 174 d after fruit collection, adult emergence was 1.0% (2021), 2.3% (2022), and 0.9% (2023) of the pupae.

### Infestation in Different Cultivars

**Fig. 3.**
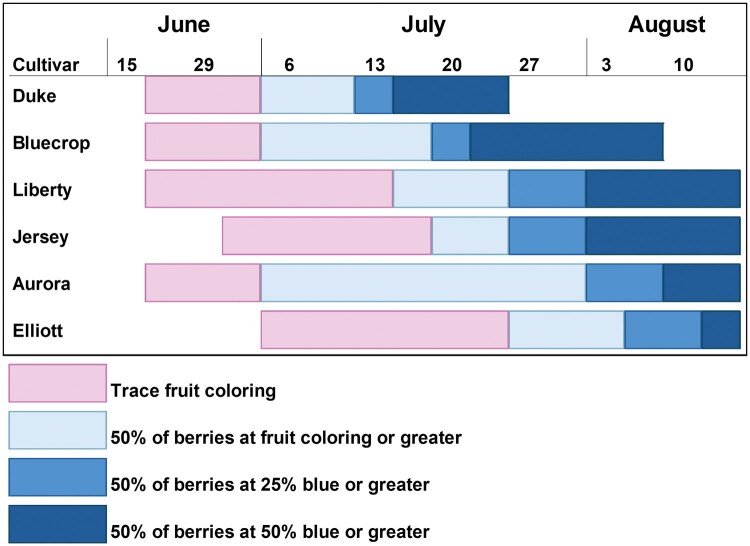
Ripening phenology of 6 highbush blueberry cultivars from a replicated planting at the Trevor Nichols Research Center in Fennville, Michigan. Berry ripeness was assessed multiple times per week each year from 2013 to 2023 and the 10-yr average sequence of ripening is displayed showing when berries were first coloring, 50% of berries at fruit coloring or greater, 50% of berries at 25% blue or greater, or when 50% of the berries were at 50% blue or greater.

Seasonal ripening phenology assessments conducted in the replicated cultivar planting at TNRC showed ‘Duke’ and ‘Bluecrop’ ripening earliest in the season, ‘Jersey’ and ‘Liberty’ having similar ripening phenologies in the middle of the season, and ‘Aurora’ and ‘Elliott’ being the latest ripening cultivars (Fig. 3). Sampling of fruit for *R. mendax* larvae in 2021 to 2023 detected more larvae in ‘Jersey’ samples collected at the end of July/early August than other cultivars in all 3 yr, a pattern that was significant in 2022 and 2023 (Table 1). The fewest larvae were detected in earlier ripening cultivars ‘Bluecrop’ (2021, 2023) and ‘Duke’ (2023), and in the latest ripening cultivar, ‘Elliott,’ in 2022. There were no significant differences among cultivars in the number of *R. mendax* reared out of blueberries for any of the ripeness categories collected on either 10 or 28 July 2023 (Table 2). On 15 August 2023, 2 to 3 times more *R. mendax* were reared out of ripening ‘Aurora’ and ‘Elliott’ berries than other cultivars and for ripe berries significantly more *R. mendax* were reared out of ‘Aurora,’ ‘Elliott,’ ‘Jersey,’ and ‘Liberty’ samples than others, as no flies were reared out of ripe ‘Bluecrop’ and ‘Duke’ samples (Table 2). In 2024, the later ripening cultivars (‘Liberty,’ ‘Jersey,’ ‘Aurora,’ ‘Elliott’) consistently had more flies reared out than the earlier ripening cultivars (‘Bluecrop,’ ‘Duke’) (Table 3). In many cases later ripening cultivars had substantially more *R. mendax* (approximately 20 to 40 per 100 g) than the early ripening cultivars (0 to approximately 9 per 100 g). Rearing data from blueberries bagged in 2023 indicated no significant differences among cultivars in the number of *R. mendax* reared out for most berry development stages on either of the 2 dates (Table 4). Only the late green fruit category had significantly more *R. mendax* reared out of ‘Liberty’ berries on 24 July 2023 than other cultivars (Table 4).

**Table 1.** The total number of *Rhagoletis mendax* per 100 g of fruit detected in ripe blueberries using filter test larval sampling. Blueberries were picked on several dates in 2021, 2022, and 2023 from 1 to 6 cultivars in a replicated unsprayed planting at the Trevor Nichols Research Center in Fennville, Michigan. Averages are presented ± SE and values with the same letters in a column are not significant at α = 0.05.

	2021		2022	2023
Cultivar	2 August	10 August	26 July	3 August
Duke	NA	NA	NA	4.8 ± 3.1 b
Bluecrop	7.9 ± 1.5 a	2.2 ± 1.1 b	18.0 ± 2.5 bc	8.4 ± 2.7 b
Liberty	13.3 ± 4.0 a	10.0 ± 1.8 ab	23.9 ± 4.5 b	19.8 ± 4.8 ab
Jersey	26.3 ± 8.8 a	15.8 ± 4.0 ab	40.4 ± 4.1 a	36.6 ± 5.3 a
Aurora	7.4 ± 2.1 a	19.5 ± 4.8 a	12.6 ± 1.9 bc	12.6 ± 2.9 ab
Elliott	15.9 ± 3.1 a	17.2 ± 4.7 ab	4.9 ± 2.5 c	13.9 ± 2.8 ab
*F*	1.78	3.36	13.58	5.26
df	4, 25	4, 23	4, 22	5, 26
*P*	0.17	0.026	<0.001	0.002

**Table 2.** The total number of *Rhagoletis mendax* per 100 g of fruit that emerged from blueberries collected from 1 to 6 cultivars and placed in rearing containers. Berries from each of 4 ripeness categories were collected on 3 different dates in 2023 from bushes in a replicated planting at the Trevor Nichols Research Center in Fennville, Michigan. Averages are presented ± SE. Significant differences among ripeness categories are indicated by upper case letters within each cultivar and significant differences among cultivar categories are indicated by lower case letters within each ripeness categories.

	10 July 2023				Ripeness statistics (within each cultivar)		
Cultivar	Late green	Trace fruit color	Ripening	Ripe fruit	H or U	df	*P*
Duke	20.0 ± 7.3	39.0 ± 17.5	24.9 ± 10.5	14.2 ± 6.6	1.5	3, 12	0.68
Bluecrop	1.7 ± 1.2 B	9.0 ± 2.8 A	22.8 ± 9.0 A	7.3 ± 1.5 A	9.4	3, 12	0.024
Liberty	33.1 ± 12.3	26.3 ± 15.8	NA	NA			
Jersey	2.5 ± 1.0	NA	NA	NA			
Aurora	2.0 ± 1.3	0.6 ± 0.6	NA	NA	10.0	1, 6	0.51
Elliott	0.5 ± 0.5	NA	NA	NA			
**Cultivar statistics (within each ripeness stage)**							
H or U	10.84	7.11	8.0	7.0			
df	5, 18	3, 12	1, 6	1, 6			
P	0.055	0.068	1.0	0.77			

	28 July 2023						
Cultivar	Late green	Trace fruit color	Ripening	Ripe fruit	H	df	*P*
Duke	NA	NA	NA	15.8 ± 5.5			
Bluecrop	NA	4.5 ± 3.3	4.5 ± 3.4	9.7 ± 3.2	2.38	2, 9	0.30
Liberty	20.1 ± 17.7	18.5 ± 7.1	37.5 ± 26.6	31.2 ± 18.6	0.30	3, 12	0.96
Jersey	13.0 ± 7.9	43.9 ± 13.8	26.7 ± 13.9	37.2 ± 8.9	4.86	3, 12	0.18
Aurora	1.0 ± 1.0 B	3.0 ± 1.8 B	11.8 ± 1.7 A	21.7 ± 10.9 A	9.98	3, 12	0.019
Elliott	0.5 ± 0.5	NA	NA	NA			
H	3.24	7.39	2.42	5.59			
df	3, 12	3, 12	3, 12	4, 15			
*P*	0.36	0.06	0.49	0.23			

	15 August 2023						
Cultivar	Late green	Trace fruit color	Ripening	Ripe fruit	H	df	*P*
Duke	NA	NA	NA	0 ± 0 b			
Bluecrop	NA	0 ± 0	0.9 ± 0.5 c	0 ± 0 b	4.36	2, 9	0.11
Liberty	NA	0.5 ± 0.5 B	8.1 ± 3.0 b AB	10.5 ± 3.5 a A	7.53	2, 9	0.023
Jersey	NA	2.4 ± 1.4	6.6 ± 1.9 b	9.4 ± 4.3 a	1.85	2, 9	0.40
Aurora	NA	4.5 ± 2.6 B	22.9 ± 3.5 a A	10.7 ± 2.4 a B	7.65	2, 9	0.022
Elliott	1.0 ± 1.0 B	0.7 ± 0.7 B	27.0 ± 11.0 a A	8.5 ± 5.2 a A	9.61	3, 10	0.022
H		6.29	14.17	15.81			
df		4, 15	4, 15	5, 17			
*P*		0.18	0.007	0.007			

**Table 3.** The total number of *Rhagoletis mendax* per 100 g of fruit that emerged from berries collected from 1 to 6 cultivars and placed in rearing containers. Berries from each of 4 ripeness categories were collected on 18 July 2024 from bushes in a replicated planting at the Trevor Nichols Research Center in Fennville, Michigan. Averages are presented ± SE. Significant differences among ripeness categories are indicated by upper case letters within each cultivar and significant differences among cultivar categories are indicated by lower case letters within each ripeness categories.

					Ripeness statistics (within each cultivar)		
Cultivar	Late green	Trace fruit color	Ripening	Ripe fruit	H	df	*P*
Duke	0 ± 0 d	0 ± 0 b	1.7 ± 1.7 c	0.3 ± 0.3 d	2.36	3, 10	0.50
Bluecrop	1.3 ± 1.3 cd B	3.1 ± 1.2 b B	8.9 ± 2.7 bc A	7.6 ± 2.6 cd AB	8.59	3, 16	0.035
Liberty	3.6 ± 1.5 bc B	21.3 ± 5.3 a A	17.5 ± 4.2 ab A	10.6 ± 2.3 bc A	9.38	3, 12	0.025
Jersey	10.9 ± 4.1 ab	19.7 ± 7.0 a	41.8 ± 16.3a	21.1 ± 7.0 abc	2.93	3, 12	0.40
Aurora	13.3 ± 4.7 a	21.3 ± 5.4 a	41.8 ± 8.0 a	29.5 ± 10.8 ab	5.56	3, 12	0.14
Elliott	2.2 ± 0.9 cd B	17.4 ± 4.1 a A	34.9 ± 8.3 a A	35.4 ± 8.1 a A	10.15	3, 12	0.017
**Cultivar statistics (within each ripeness stage)**							
H	15.91	15.92	14.61	14.56			
df	5, 18	5, 19	5, 18	5, 19			
*P*	0.007	0.007	0.012	0.012			

**Table 4.** The total number of *Rhagoletis mendax* per 100 g of fruit that emerged from berries collected in 2023 from 1 to 6 cultivars and placed in rearing containers. Blueberry canes were covered with mesh paint strainer bags after bloom to prevent *R. mendax* oviposition. Mesh bags were subsequently removed on 1 of 2 dates (24 July 2023, 31 July 2023) to allow *R. mendax* adults in the field to lay eggs on the berries. All fruit were collected after 1 wk of exposure and were placed in rearing containers after separation into 1 of 4 ripeness categories. Averages are presented ± SE. Significant differences among ripeness categories are indicated by upper case letters within each cultivar and significant differences among cultivar categories are indicated by lower case letters within each ripeness categories

	Bag removal date:						
	24 July 2023				Ripeness statistics (within each cultivar)		
Cultivar	Late green	Trace fruit color	Ripening	Ripe fruit	H or U	df	*P*
Duke	NA	NA	NA	0 ± 0			
Bluecrop	0 ± 0 c	5.5 ± 5.5	15.3 ± 15.3	1.1 ± 1.1	0.68	3, 11	0.68
Liberty	10.9 ± 2.8 a	16.0 ± 5.9	7.0 ± 7.0	11.8 ± 9.2	1.39	3, 12	0.71
Jersey	3.5 ± 2.0 bc	7.1 ± 4.7	NA	14.2 ± 12.5	0.29	2, 9	0.87
Aurora	6.5 ± 3.0 ab	16.0 ± 6.4	NA	NA	3.5	1, 6	0.19
Elliott	0.8 ± 0.8 c	5.5 ± 5.5	NA	NA	7.5	1, 6	0.85
**Cultivar statistics (within each ripeness stage)**							
H or U	15.42	2.85	5.0	6.19			
Df	5, 17	4, 15	1, 4	3, 11			
*P*	0.009	0.58	0.80	0.10			

	31 July 2023						
Cultivar	Late green	Trace fruit color	Ripening	Ripe fruit	H or U	df	*P*
Duke	NA	NA	NA	0 ± 0			
Bluecrop	NA	0 ± 0	0 ± 0	0 ± 0	0	2, 6	1.0
Liberty	2.2 ± 1.3	4.2 ± 4.2	0 ± 0	0 ± 0	3.85	3, 12	0.28
Jersey	0 ± 0	4.1 ± 2.9	0 ± 0	2.5 ± 2.5	3.73	3, 11	0.29
Aurora	3.5 ± 3.5	5.2 ± 4.4	8.7 ± 8.7	1.3 ± 1.3	0.71	3, 12	0.87
Elliott	0 ± 0	0 ± 0	NA	NA	8.0	1, 6	1.0
H	3.85	4.71	3.67	3.17			
Df	3, 12	4, 15	3, 10	4, 15			
*P*	0.28	0.32	0.30	0.53			

### Fruit Ripeness Preference

Collection of blueberries at the 4 different ripeness categories in 2023 showed that *R. mendax* is capable of laying eggs in berries at all stages of ripeness, including late green fruit (Table 2). Not all cultivars had significant differences in the number of *R. mendax* reared out among different ripeness categories, but where there were we found more flies emerging from berries in the ripening and ripe fruit categories than in the late green or fruit coloring (Table 2). A similar pattern was found in 2024, with *R. mendax* emerging out of berries at all ripeness stages but more emerging from berries in the ripening and ripe categories (Table 3). When blueberries were exposed to wild flies for 1 wk, *R. mendax* was able to lay eggs in berries at any ripeness stage (Table 4). There were no significant differences among ripeness categories in the number of *R. mendax* reared out for any of the cultivars, but significantly more *R. mendax* were reared out of berries that were exposed beginning on 24 July 2023 than those exposed on 31 July 2023 (late green: *U* = 100.5, df = 1, 21, *P* = 0.022; trace color: *U* = 268.0, df = 1, 38, *P* = 0.034, ripening: *U* = 32.0, df = 1, 16, *P* = 0.09, ripe: *U* = 163.0, df = 1, 28, *P* = 0.01).

## Discussion

This study provides insights into the current phenology and host associations of *R. mendax* in relation to highbush blueberry production in Michigan where it remains a key pest, despite the invasion by *D. suzukii*. Previous studies have shown *R. mendax* phenology is flexible, with populations shifting emergence earlier or later in the season, depending on when ripe host fruit are available (Lathrop and Nickels 1932, Teixeira and Polavarapu 2001). Shope et al. (2023) recently found evidence for a shift to later phenology of *R. mendax* in New Jersey blueberry farms, a trend the authors suggest may be related to increasing competition from the recently established *D. suzukii.* That region had previously reported selection to later activity by *R. mendax* (Teixeira and Polavarapu 2003), indicating a propensity of the population to adapt in this way. In this study we found evidence for a shift of *R. mendax* adult fly activity to earlier in the season, a trend supported by data from rearing samples (Table 2). This shift could be partly due to the replacement of a slightly later ripening cultivar (‘Rubel’) with a slightly earlier ripening cultivar (‘Bluecrop’) when the field was replanted in spring 2010. However, our sampling at unsprayed and minimally sprayed blueberry fields around the region also suggests activity earlier in the season with *R. mendax* larvae being detected even in the earliest ripening samples, a trend that has become more pronounced in recent years (Fig. 1). This shift may be due to increased competition from *D. suzukii* later in the summer, as the larvae of this co-occurring fruit pest are competitive (Shope et al. 2023), although mechanisms of competition between the 2 species have yet to be explored. In these minimally sprayed fields, infestation by *D. suzukii* larvae was lower earlier in the season (early-mid July), but increased to high levels later in the season (mid-late August–September) (Fig. 1). Similar trends have been found in previous studies in highbush blueberry in this region (Leach et al. 2019, Van Timmeren et al. 2025). Under this scenario, *R. mendax* adults emerging and laying eggs while berries are starting to ripen will have less competition for oviposition resources than those emerging later. To more fully test this hypothesis would require a comparison of *R. mendax* infestation patterns in years before and after *D. suzukii* establishment. The adult fly trapping data provide further evidence for variability in the population over multiple years. The very low capture of adults in 2010 and 2011 coincided with newly planted blueberry bushes not yet producing fruit, whereas the low captures from 2014 to 2019 coincided with plenty of unsprayed fruit being available. It is possible this decrease was due to competition with *D. suzukii*, however, other studies have found variability in adult capture and emergence from 1 yr to the next (Lathrop and Nickels 1932, Shope et al. 2023, Drummond et al. 2025) so it not possible to assign a cause to this decline. Additionally, while *Rhagoletis* is known to visit alternative host plants (Hood et al. 2012) it is unlikely that the flies caught in our traps included many *R. pomonella* adults as additional yellow sticky traps located in a nearby apple orchard caught adults much later in the season (peak capture in mid/late-August; Van Timmeren and Isaacs, unpublished data).

The shift of *R. mendax* activity to earlier in the season in Michigan is also supported by rearing flies from blueberries at different ripeness stages. As expected, *R. mendax* were more often reared from ripening and ripe berries than late green and trace color blueberries. Despite this, *R. mendax* were still consistently reared from late green and trace color blueberries, including some instances where substantial numbers were reared from the late green stage (see ‘Liberty’ data in Table 2). Given that the blueberry samples were picked from bushes and placed in rearing containers rather than being allowed to ripen naturally, it is entirely possible this method underestimated *R. mendax* infestation in unripe berries due to the lower quality of the host compared to berries ripening on the bushes. If undisturbed, larvae in unripe or early ripening berries would experience changing host quality as the berry ripens around them. Given the shorter time frame in which we conducted rearing experiments (2023 to 2024) it is possible that *R. mendax* have always laid eggs in green and early ripening blueberries and this behavior has not been previously documented. Alternatively, adult flies may have shifted to laying eggs in less ripe fruit as a way to avoid competition with *D. suzukii*. Additional research is needed to examine the relationship between oviposition, fruit ripening, and competition with *D. suzukii* in Michigan, with potential to compare patterns across the geographic range where these 2 species are now co-occurring.

Our results provide insights for managing blueberry fields in Eastern North America where growers typically wait until the berries ripen before protecting against *D. suzukii* (Rodriguez-Saona et al. 2019). Since *R. mendax* are capable of laying eggs in green and early ripening blueberries it will remain important to include monitoring for adult flies to determine when they become active rather than using fruit ripeness as a guide for timing insecticide applications. In growing seasons where *D. suzukii* activity is delayed due to winter and spring weather (Leach et al. 2019), fields with monitoring traps that catch zero *R. mendax* can be places where sprays are delayed and production costs are reduced. This will be increasingly important as biological control from adventive or released species of figitid wasps (Rossi-Stacconi et al. 2025) becomes an increasingly important component of integrated pest management programs for *D. suzukii*.

In addition to the shift in *R. mendax* adult flight and oviposition phenology, we detected more *R. mendax* larvae in ‘Jersey’ blueberries and more flies reared out of later ripening cultivars such as ‘Jersey’ and ‘Liberty.’ These results are similar to those reported by Liburd et al. (1998) where later ripening cultivars had more *R. mendax* larvae. However, the earliest ripening cultivars in this study (‘Duke’ and ‘Bluecrop’) begin ripening in late June and early July at this site (Fig. 3) and the earliest sampling conducted in this study (10 July 2023, Table 2) already had substantial *R. mendax* infestation. Given the trend of oviposition in berries that are still green or showing trace color, it is possible that the lower infestation detected on later sampling dates (mid-July to mid-August) indicates that larvae had already matured and infested berries had dropped off the bush by the time later fruit samples were collected. Also, ripe berries collected from early cultivars on later sampling dates were likely to have been declining in quality and may have been less attractive as an oviposition resource. Liburd et al. (1998) found some evidence for this in their sampling and similar results have been found for *R. pomonella* (Reissig 1974). Blueberries ripen in an asynchronous manner, with berries of various ripening stages present on the bush for weeks in July and August. Combined with the variation in ripening timing among cultivars and increasing competition from *D. suzukii* late in the season, this creates a complex environment with multiple interacting factors that can affect *R. mendax* phenology. Studies to compare cultivars with the full range of ripening timings provided by modern breeding programs may identify blueberry cultivars that can avoid periods of infestation, however the plasticity of *R. mendax* phenology may result in only temporary relief from pest risk and we expect the local selective forces to vary across the geographic range of *R. mendax*.

By monitoring emergence of *R. mendax* from samples of pupae we found that ∼1% of pupae emerged as non-diapausing adults in the same growing season, confirming the brief descriptions in previous studies (Payne and Berlocher 1995, Dambroski and Feder 2007). This result is similar to the reports from other species within the *Rhagoletis* genus, including *R. completa* (18%: Rull et al. 2019), *R. indifferens* (∼0.4% to 1.1%: Frick et al. 1954), *R. lycopersella* (58%: Smyth 1960), *R. pomonella* (∼5% to 60%: Prokopy 1968, Dolphin et al. 1970, Dambroski and Feder 2007, Yee et al. 2021, McIntyre et al. 2023), and *R. zephyria* (1.3% to 1.9%: Yee et al. 2021), suggesting there is standing genetic variation that can support shifts in phenology of *Rhagoletis* without a need for mutation and selection to adjust activity timing. While we did find emergence of non-diapausing adults when pupae were placed in an environmental chamber, there was no evidence of such activity in any of the larval sampling or rearing samples we collected from field sites. Instead, all indications pointed to adults emerging in late-June/early-July and activity declining in August. Given the low percentage of adults emerging as non-diapausing adults, it may not be possible to detect such emergence in the field without more focused, intense sampling. Pupae in this study were placed in an environmental chamber set to a constant temperature and day length, which may not be optimal for non-diapausing individuals. Either way, this plasticity in adult emergence points to the need for blueberry growers to be vigilant as local populations of *R. mendax* could have a longer period of activity driven by earlier activity to escape competition from *D. suzukii* and the insecticides used to control them. At the same time, the use of later-ripening cultivars grown in a future environment with longer growing seasons may select for a second generation of *R. mendax* from the non-diapausing flies. Future monitoring for this pest and its natural enemies should continue across the widening harvest period of blueberries to determine how the insect community adapts to changing host availability. Forbes et al. (2009) and Hood et al. (2015) have highlighted that the parasitoid community on *Rhagoletis* can shift in response to their host larvae, and we would expect changing phenology of this fruit fly to also select for parasitoids that can exploit this resource.

In summary, in this study, we found that *R. mendax* populations in Michigan have shifted activity to earlier in the growing season, and adults are laying eggs in unripe and early ripening blueberries in addition to the later ripening and ripe berries that this pest is expected to oviposit into. Larvae hatching from eggs laid in the unripe fruit can survive to pupation, resulting in infestation risk and potential selection for earlier active *R. mendax*. We found some evidence for cultivar preferences, but additional research is needed to further elucidate the relationship between adult *R. mendax* emergence and cultivar ripening. Taken together, these findings can be used to support the development and implementation of IPM programs for blueberry production to minimize the economic impact of a key native fly pest during the harvest season, and to support the expanding biological control program for the invasive *D. suzukii*.
